# Effect of Adjuvant Silodosin on Stone Clearance After Extracorporeal Shock Wave Lithotripsy for Renal Stones: A Randomised Controlled Trial

**DOI:** 10.3390/jcm15072471

**Published:** 2026-03-24

**Authors:** Phanpon Leelahawong, Chinnakhet Ketsuwan

**Affiliations:** 1 Department of Surgery, Rayong Hospital, Rayong 21000, Thailand; phanpon@hotmail.com; 2 Division of Urology, Department of Surgery, Faculty of Medicine Ramathibodi Hospital, Mahidol University, Bangkok 10400, Thailand

**Keywords:** silodosin, extracorporeal shock wave lithotripsy, renal stones, stone-free rate, medical expulsive therapy

## Abstract

**Background/Objectives**: To evaluate whether adjunctive silodosin improves the stone-free rate (SFR) and clinical outcomes of extracorporeal shock wave lithotripsy (ESWL) for renal calculi. **Methods**: In this prospective randomised controlled trial, 100 adults with solitary radiopaque non-lower pole renal stones measuring 5–20 mm underwent single-session ESWL and were randomised (1:1) to receive either silodosin 8 mg once daily plus standard care or standard care alone for up to 12 weeks. Participants were followed up for three months. The primary outcome was SFR at three months on follow-up imaging. The secondary outcomes included time to stone clearance, renal colic episodes, analgesic requirement and adverse events. **Results**: At three months, the SFR was higher in the silodosin group than in the control group (68.0% vs. 50.0%; RR 1.36, 95% CI 0.97–1.90), but this difference did not reach statistical significance (*p* = 0.067). In a prespecified exploratory subgroup analysis, patients with stones measuring 10–20 mm showed a higher SFR with silodosin than controls (61.8% vs. 34.4%; *p* = 0.026), whereas no benefit was observed for stones measuring 5–9 mm (*p* = 0.803). Time-to-clearance analysis using Kaplan–Meier methods suggested earlier confirmed stone clearance in the silodosin group (hazard ratio 1.58, 95% CI 1.02–2.45; log-rank *p* = 0.036). Silodosin was also associated with fewer renal colic episodes and lower analgesic requirements. No serious drug-related adverse events were observed. **Conclusions**: This randomised controlled trial did not meet its primary endpoint because adjunctive silodosin did not significantly improve the overall SFR after ESWL. However, a possible benefit was observed in patients with renal stones measuring 10–20 mm, together with improved pain-related outcomes. These findings suggest that silodosin may have a role in selected patients, but the subgroup effects should be considered hypothesis-generating rather than definitive.

## 1. Introduction

Nephrolithiasis affects approximately 1–13% of the global population and imposes a substantial healthcare burden [1]. Management of symptomatic renal stones often requires a combination of procedural and adjunctive medical strategies. Extracorporeal shock wave lithotripsy (ESWL) remains a widely used non-invasive treatment for uncomplicated renal and ureteric calculi measuring < 20 mm because it has a more favourable safety profile and a higher patient acceptance than other treatment modalities [2]. Nevertheless, incomplete clearance of fragmented calculi following ESWL is common, with residual fragments reported in up to half of treated patients. These fragments may result in recurrent pain, infection and the need for secondary interventions, highlighting the clinical importance of optimising post-ESWL fragment passage.

Ureteric peristalsis is mediated in part by α1-adrenergic receptors, and pharmacological blockade of these receptors reduces ureteric smooth muscle tone and intraluminal pressure, thereby facilitating stone transit [3]. Silodosin, a highly selective α1A-adrenoceptor antagonist, has demonstrated efficacy as a medical expulsive therapy for ureteric stones [4] and has been proposed as a potential adjunct to enhance fragment clearance after ESWL. However, evidence supporting the routine use of α-blockers following ESWL remains inconsistent [2,5,6,7], particularly in patients with renal stones, and the characteristics of patients likeliest to benefit have not been clearly defined. Accordingly, this randomised controlled trial was designed to evaluate whether adjunctive silodosin improves stone-free rates and clinical outcomes following ESWL for renal calculi.

## 2. Materials and Methods

### 2.1. Ethical Approval and Patient Consent

This study was conducted according to the principles of the Declaration of Helsinki. The study protocol was approved by the institutional ethics committee (protocol code: RYH REC No. E009/2566, approval date: 21 April 2023) and was registered with the Thai Clinical Trials Registry (TCTR20230502002). Written informed consent was obtained from all participants.

### 2.2. Study Design and Patients

This prospective randomised controlled trial was conducted at Rayong Hospital, Thailand, between May 2023 and March 2024.

#### Inclusion and Exclusion Criteria

Eligible participants were adults aged ≥18 years with a solitary radiopaque renal stone measuring 5–20 mm located in the renal pelvis, upper calyx, or mid-calyx. The diagnosis was confirmed by non-contrast computed tomography (NCCT). The exclusion criteria included lower-pole stones, radiolucent stones, indwelling ureteric stents or nephrostomy tubes, active urinary tract infection, renal impairment (eGFR < 60 mL/min/1.73 m^2^), coagulopathy, pregnancy, aortic- or renal-artery aneurysm, significant skeletal deformity, high-grade hydronephrosis and concurrent use of α-blockers or calcium channel blockers. Stone size was measured as the maximal diameter on multi-planar reconstructed coronal images from NCCT.

### 2.3. Experimental Methods

**Figure 1:**
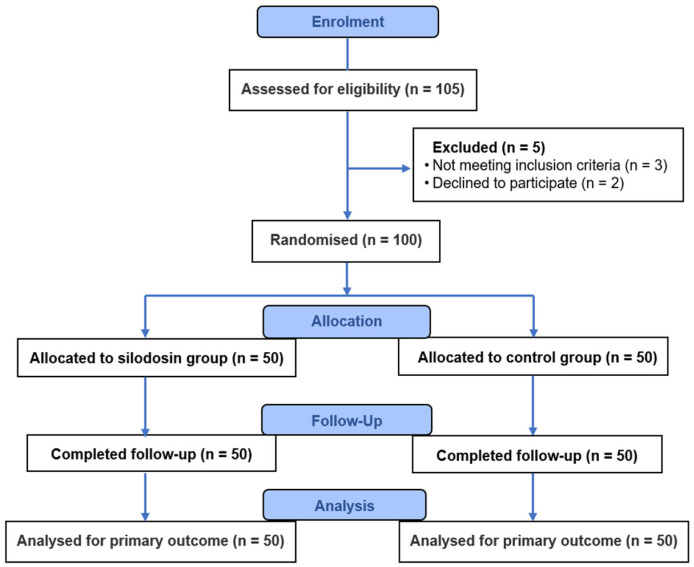
CONSORT flow diagram of patient enrolment, randomisation, follow-up and analysis.

Participants were randomly assigned in a 1:1 ratio to receive either silodosin or standard care using computer-generated block randomisation. A total of 100 patients were enrolled and randomised to the silodosin group (*n* = 50) or the control group (*n* = 50) (Figure 1). The randomisation sequence was generated by an independent investigator who was not involved in patient enrolment, with concealed block sizes. Allocation concealment was ensured using sequentially numbered, opaque, sealed envelopes. Radiological-outcome assessment was performed by assessors blinded to the treatment allocation. Due to the absence of a placebo, the patients and treating physicians were aware of the group assignments. Randomisation was not stratified by stone size; stone-size subgroup analyses were pre-specified in the statistical-analysis plan.

All participants underwent ESWL using a Siemens Modularis Vario Lithostar electromagnetic lithotripter (Siemens Healthineers, Erlangen, Germany). All procedures were performed by experienced urologists using a standardised institutional protocol. The protocol included energy ramping, a shock frequency of 60–90 shocks per minute, and a maximum of 3000–5000 shocks delivered under fluoroscopic guidance. Treatment was terminated earlier, at the discretion of the operating physician, if adequate fragmentation was judged fluoroscopically, consistent with routine clinical practice. Standardised procedural analgesia was administered to all patients to ensure consistency across both study arms.

The patients in the intervention group received oral silodosin 8 mg once daily starting on the day of ESWL and continued until stone clearance, transition to a secondary intervention or for a maximum of 12 weeks. The control group received standard care consisting of hydration advice and analgesics on demand, without α-blockers. Treatment adherence to silodosin was assessed through pill counts and review of prospective patient medication diaries at each follow-up visit.

Follow-up visits were conducted at 2 weeks, 1 month, 2 months and 3 months post-ESWL. The evaluations included fragment passage, renal colic episodes, analgesic use and adverse effects. Renal colic episodes were defined as episodes of acute flank pain consistent with renal colic requiring medical consultation or analgesic treatment. These events were recorded in patient diaries and verified during scheduled follow-up visits. Analgesic use was recorded prospectively in patient diaries, and cumulative analgesic consumption was calculated as the total milligrams of diclofenac taken during the 12-week follow-up period. Imaging consisted of abdominal ultrasonography and kidney–ureter–bladder (KUB) radiography at each visit. NCCT was reserved for patients with equivocal imaging findings or persistent symptoms.

### 2.4. Outcome Measures

The primary outcome was the stone-free rate (SFR), defined as the complete absence of residual fragments or the presence of clinically insignificant fragments (<4 mm) on follow-up imaging at three months. For equivocal KUB/ultrasound findings or when the fragment size could not be reliably determined (particularly for fragments near the 4 mm threshold), NCCT was performed to adjudicate stone-free status. The fragment size was assessed according to the imaging modality used at follow-up. Patients who required secondary intervention during the 12-week follow-up period were classified as treatment failures and were therefore considered not stone-free in the primary analysis.

The secondary outcomes included time to stone clearance (weeks from ESWL to confirmed clearance), number of renal colic episodes, total analgesic consumption (cumulative diclofenac dose), steinstrasse formation, occurrence of secondary interventions (repeat ESWL, ureteroscopy, or ureteric stenting), and drug-related adverse events.

### 2.5. Sample Size

Sample size estimation was based on the expected stone-free rate (SFR) after ESWL. Previous studies have reported SFRs of approximately 50–60% with standard care [8,9], whereas trials evaluating α-blockers as adjunctive therapy have suggested absolute improvements of 15–25% in selected populations [10,11]. Based on these data, an SFR of 50% was assumed for the control group and an anticipated improvement to 70% in the silodosin group.

Under standard two-proportion assumptions with a two-sided α level of 0.05 and 80% power, approximately 93 participants per group would be required to detect this difference. However, as this was a single-centre trial conducted within a defined recruitment period, a total sample size of 100 participants (50 per group) was considered feasible. Consequently, the study had limited statistical power to detect modest differences in the primary endpoint, and the findings should be interpreted with caution.

### 2.6. Statistical Analysis

All statistical analyses were performed using STATA version 14.1 (StataCorp, College Station, TX, USA). Continuous variables were assessed for normality and summarised as means ± standard deviations (SDs), and categorical variables were presented as frequencies and percentages. Between-group comparisons were conducted using independent *t*-tests for normally distributed continuous variables or non-parametric tests (as appropriate) and Fisher’s exact tests or χ^2^ tests (as appropriate) for categorical variables. To explore whether the treatment effect differed according to baseline stone size, a treatment-by-stone-size interaction term was tested using logistic regression.

Time-to-event outcomes were analysed using the Kaplan–Meier method. Time to stone clearance was defined as the interval from ESWL to the first follow-up visit at which stone-free status was confirmed on imaging (scheduled at 2 weeks, 1 month, 2 months, and 3 months). Patients without confirmed stone clearance during follow-up were censored at their last assessment. Comparisons between groups were performed using the log-rank test, and hazard ratios (HRs) with 95% confidence intervals (CIs) were estimated using a Cox proportional hazards model.

To assess the robustness of the primary endpoint in relation to the mixed imaging modalities used for follow-up, a sensitivity analysis restricted to patients who underwent NCCT at the 3-month follow-up was performed.

All analyses were conducted on an intention-to-treat basis. A two-sided *p* value < 0.05 was considered statistically significant. The CONSORT 2025 checklist is provided in the Supplementary Materials [12].

## 3. Results

### 3.1. Demographic and Clinical Characteristics

No statistically significant differences were observed between the silodosin and control groups in baseline demographic characteristics, stone parameters, or ESWL-related variables (Table 1).

### 3.2. Primary and Secondary Outcomes

At three months, stone-free status was achieved in 68.0% (34/50) of patients in the silodosin group and 50.0% (25/50) in the control group (RR 1.36, 95% CI 0.97–1.90; *p* = 0.067) (Table 2). Stone-free status at follow-up was primarily assessed using KUB radiography and ultrasonography. NCCT was performed when findings were equivocal or when persistent symptoms required confirmation. At the 3-month assessment, NCCT was performed in 12 patients in the silodosin group and 14 patients in the control group, indicating a broadly similar distribution between study arms. In a sensitivity analysis restricted to patients who underwent NCCT at the 3-month follow-up, stone-free status was observed in 7/12 patients (58.3%) in the silodosin group and 6/14 patients (42.9%) in the control group (RR 1.36, 95% CI 0.63–2.94). The direction of the treatment effect remained consistent with that observed in the primary analysis, although the estimate was imprecise because of the small number of CT-confirmed cases.

**Figure 2:**
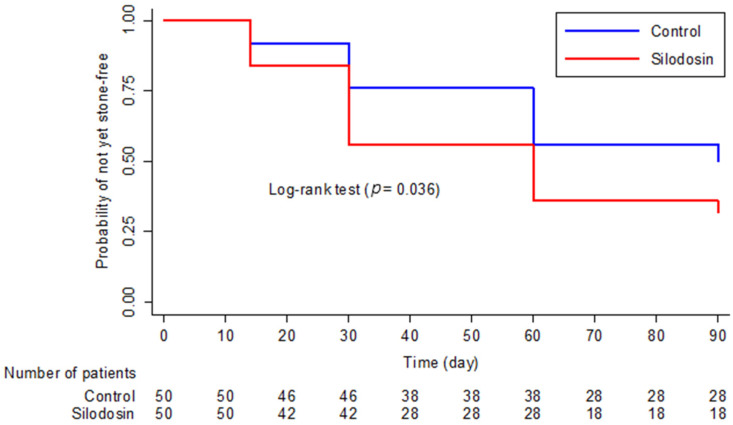
Kaplan–Meier curves for time to stone clearance after extracorporeal shock wave lithotripsy.

Kaplan–Meier analysis demonstrated a higher probability of earlier stone clearance in the silodosin group compared with the control group (log-rank *p* = 0.036), corresponding to a hazard ratio of 1.58 (95% CI 1.02–2.45) (Figure 2).

Acute renal or ureteric colic occurred less frequently in the silodosin group than in the control group (10.0% vs. 26.0%; RR 0.38, 95% CI 0.15–0.97; *p* = 0.037). The cumulative analgesic requirement was also lower in the silodosin group (265.0 ± 55.6 mg vs. 321.9 ± 65.9 mg; mean difference −56.9 mg, 95% CI −80.5 to −33.3; *p* = 0.001). In addition, fewer patients required additional analgesia compared with controls (12.0% vs. 30.0%; RR 0.40, 95% CI 0.17–0.94; *p* = 0.027).

Steinstrasse developed in two patients (4.0%) in the silodosin group and three patients (6.0%) in the control group (RR 0.67, 95% CI 0.12–3.74; *p* = 0.646). Secondary interventions during the 12-week follow-up period were required in 18.0% of patients in the silodosin group and 30.0% in the control group (RR 0.60, 95% CI 0.29–1.25; *p* = 0.160), primarily due to persistent symptoms, steinstrasse, or lack of fragment progression on follow-up imaging.

No serious ESWL-related or medication-related adverse events were observed during the study period. One patient in the silodosin group experienced retrograde ejaculation, which resolved spontaneously after discontinuation of the study medication (Table 3).

In a pre-specified subgroup analysis stratified by stone size, a higher stone-free rate was observed in the silodosin group among patients with stones measuring 10–20 mm compared with the control group (61.8% [21/34] vs. 34.4% [11/32]; RR 1.80, 95% CI 1.07–3.01; *p* = 0.026). In contrast, no significant difference was observed among patients with stones measuring 5–9 mm (81.3% [13/16] vs. 77.8% [14/18]; RR 1.04, 95% CI 0.70–1.54; *p* = 0.803).

A treatment-by-stone-size interaction analysis was performed using logistic regression. The interaction term was not statistically significant (*p* for interaction = 0.10). Therefore, the subgroup findings are presented as exploratory.

## 4. Discussion

In this randomised controlled trial, adjunctive silodosin after ESWL did not achieve a statistically significant improvement in the primary endpoint of overall SFR compared with standard care, although a numerical trend towards higher clearance was observed. While α-blockers after ESWL have been investigated previously, contemporary randomised trials specifically evaluating silodosin in a well-defined renal stone population remain relatively limited. In our study, a possible size-related trend was observed among patients with stones measuring 10–20 mm, accompanied by improvements in pain-related outcomes.

Stone clearance following ESWL is a multifactorial process influenced by stone-related characteristics (size, density, number and location) as well as patient and procedural factors, including body habitus, skin-to-stone distance and shock-wave parameters [4,5]. As these variables were well balanced between the groups in the present study, the observed differences are unlikely to be attributable to procedural confounding and more plausibly reflect the pharmacodynamic effects of silodosin.

α-blockers enhance post-ESWL stone clearance primarily by promoting ureteric−smooth muscle relaxation and facilitating the passage of residual fragments rather than by influencing initial stone fragmentation [11,13,14,15,16]. This mechanism is particularly relevant for larger residual fragments (≥10 mm), which are less likely to pass spontaneously [11,17]. Accordingly, the greater efficacy observed in patients with stones measuring 10–20 mm in the present study is biologically plausible. However, this finding should be interpreted with caution because it arose from a pre-specified but underpowered subgroup analysis and should be considered hypothesis-generating rather than confirmatory. Importantly, the study was not powered to detect interaction effects between stone size and treatment response, and no adjustment was made for multiple comparisons.

The observed size-dependent trend is consistent with the improved post-ESWL outcomes with α-blockers in selected patient populations reported in previous studies. Gravina et al. demonstrated a significantly higher ESWL success rate (81% Vs. 55%) for renal stones measuring 10–20 mm when tamsulosin was administered at 90 days [18]. Conversely, De Nunzio et al. reported no benefit from α-blocker therapy after ESWL in an unselected cohort [8]. Differences in stone size distribution, imaging modality for outcome assessment and follow-up duration may partially explain these discrepant findings. In particular, the inclusion of smaller stones—which often clear spontaneously—may dilute treatment effects when heterogeneous populations are analysed.

Compared with non-selective α-blockers, silodosin exhibits high selectivity for the α1A-adrenoceptor subtype, which is prominently expressed in the lower urinary tract, including the distal ureter and bladder neck. This selectivity results in pronounced ureteric relaxation, reduced intraluminal pressure and smoother fragment transit. In addition, the inhibition of calcium influx in ureteric smooth muscle may reduce ureteral spasm and nociceptive C-fibre activation, thereby alleviating pain. These mechanisms likely explain the observed reductions in renal-colic episodes and analgesic requirements in the silodosin group, findings that are consistent with those of prior studies that evaluated α-blockers following ESWL [19,20,21]. However, these pain-related outcomes should be interpreted in the context of an unblinded study design and their subjective nature.

Although silodosin did not significantly reduce the incidence of steinstrasse or the need for secondary interventions in the present study, the overall complication rates were low and comparable between the groups. The study was not powered to detect differences in uncommon adverse events; therefore, conclusions regarding complication prevention should be drawn cautiously.

Silodosin was generally well tolerated, with no serious cardiovascular or drug-related adverse events observed. While mild and reversible ejaculatory dysfunction occurred in only one patient (2.0%), the sample size was insufficient to fully evaluate the safety profile of silodosin, particularly for rare adverse effects.

The present study has several limitations. First, although this was a prospective randomised trial, the study was conducted at a single centre with a relatively modest sample size, which may limit the generalisability of the findings. In addition, the achieved statistical power was limited for detecting modest differences in stone-free rates between groups. Consequently, the relatively wide confidence interval around the treatment effect suggests that the results should be interpreted cautiously and may be considered inconclusive rather than definitively negative. Second, although subgroup analyses according to stone size were pre-specified, the relatively small number of patients within each subgroup increases the risk of chance findings and type I error. Therefore, these subgroup results should be interpreted as exploratory and hypothesis-generating. Third, baseline stone size was measured using NCCT, whereas follow-up evaluation relied primarily on ultrasonography and KUB radiography, with selective use of NCCT in equivocal cases. This pragmatic approach reflects routine clinical practice but may influence the apparent stone-free rates, introduce the possibility of verification bias, and limit direct comparison with studies that use uniform CT-based outcome assessment. To explore the robustness of the primary endpoint, we performed a sensitivity analysis restricted to patients who underwent NCCT at the 3-month follow-up. Although the number of CT-confirmed cases was limited, the direction of the treatment effect was consistent with that observed in the primary analysis, but the estimate remained imprecise due to the small sample size. Despite these limitations, several methodological strengths should be noted, including the prospective randomised design, allocation concealment, blinded radiological outcome assessment, and the use of a standardised ESWL protocol, all of which strengthen the internal validity of the study.

## 5. Conclusions

In summary, adjunctive silodosin after ESWL did not significantly improve the overall SFRs in the present study but was associated with meaningful reductions in pain and analgesic use. A potential size-dependent benefit was observed in patients with stones measuring 10–20 mm, but this finding should be regarded as exploratory. Routine post-ESWL use of silodosin is not supported by the present data, but selective use in carefully chosen patients may be considered pending confirmation in adequately powered multicentre trials.

## Figures and Tables

**Table 1:** Baseline participant characteristics and operative parameters.

Variable	Silodosin (*n* = 50)	Control (*n* = 50)	*p*
Age (years), mean (SD)	50.9 ± 11.9	53.9 ± 12.6	0.226 *
Gender, *n* (%)			
Male	27 (54.0)	28 (56.0)	0.841 **
Female	23 (46.0)	22 (44.0)	
BMI (kg/m^2^), mean (SD)	26.7 ± 4.0	25.3 ± 4.6	0.100 *
Stone laterality, *n* (%)			
Left	21 (42.0)	25 (50.0)	0.422 **
Right	29 (58.0)	25 (50.0)	
Stone location, *n* (%)			
Upper calyx	6 (12.0)	5 (10.0)	0.820 ***
Mid calyx	31 (62.0)	34 (68.0)	
Renal pelvis	13 (26.0)	11 (22.0)	
Stone size, mm, mean (SD)	12.8 ± 6.9	12.6 ± 7.1	0.994 *
Stone density, HU, mean (SD)	808.7 ± 152.7	808.9 ± 161.5	0.137 *
Number of shock waves, mean (SD)	3740.0 ± 632.7	3600.0 ± 580.3	0.252 *
Treatment duration, minutes, mean (SD)	60.2 ± 6.1	61.1 ± 6.2	0.466 *

* Student’s *t*-test; ** Chi-square test; *** Fisher’s exact test. Abbreviations: BMI: body mass index; HU: Hounsfield units; SD: standard deviation.

**Table 2:** Study outcomes.

Outcome	Silodosin (*n* = 50)	Control (*n* = 50)	Effect Estimate (95% CI)	*p*
Stone-free rate, *n* (%)	34 (68.0)	25 (50.0)	RR 1.36 (0.97 to 1.90)	0.067 **
Steinstrasse, *n* (%)	2 (4.0)	3 (6.0)	RR 0.67 (0.12 to 3.74)	0.646 ***
Acute colic episodes, *n* (%)	5 (10.0)	13 (26.0)	RR 0.38 (0.15 to 0.97)	0.037 **
Cumulative analgesic dosage, mg, mean (SD)	265.0 ± 55.6	321.9 ± 65.9	Mean difference −56.9 mg (−80.5 to −33.3)	0.001 *
Additional analgesia required, *n* (%)	6 (12.0)	15 (30.0)	RR 0.40 (0.17 to 0.94)	0.027 **
Secondary intervention, *n* (%)	9 (18.0)	15 (30.0)	RR 0.60 (0.29 to 1.25)	0.160 **
ESWL	6	10	—	—
URS	2	3	—	—
Ureteric stent	1	2	—	—
Imaging modality used for 3-month SFR assessment				
US/KUB only, *n* (%)	38 (76.0)	36 (72.0)	—	—
NCCT performed, *n* (%)	12 (24.0)	14 (28.0)	—	—

* Student’s *t*-test; ** Chi-square test; *** Fisher’s exact test. Abbreviations: ESWL: extracorporeal shock wave lithotripsy; SD: standard deviation; URS: ureteroscopy; US: ultrasonography; KUB: kidney–ureter–bladder radiography; NCCT: non-contrast computed tomography.

**Table 3:** Adverse events.

Adverse Event	Silodosin (*n* = 50)	Control (*n* = 50)
Retrograde ejaculation	1 (2.0)	0 (0.0)
Dizziness	0 (0.0)	0 (0.0)
Hypotension	0 (0.0)	0 (0.0)
Drug discontinuation due to adverse events	0 (0.0)	0 (0.0)
Serious adverse events	0 (0.0)	0 (0.0)

## Data Availability

The data supporting the findings of this study are available from the corresponding author upon reasonable request. Due to privacy and ethical restrictions, the dataset is not publicly accessible.

## Supplementary Materials

The following supporting information can be downloaded at: https://www.mdpi.com/article/10.3390/jcm15072471/s1, CONSORT 2025 checklist item description. Reference [12] is cited in Supplementary Materials.

## Abbreviations

The following abbreviations are used in this manuscript:

α1A	Alpha-1A adrenergic receptor
BMI	Body mass index
CI	Confidence interval
CONSORT	Consolidated Standards of Reporting Trials
CT	Computed tomography
eGFR	Estimated glomerular filtration rate
ESWL	Extracorporeal shock wave lithotripsy
HU	Hounsfield units
IRB	Institutional Review Board
KUB	Kidney–ureter–bladder radiography
MET	Medical expulsive therapy
NCCT	Non-contrast computed tomography
RCT	Randomised controlled trial
RR	Relative risk
SD	Standard deviation
SFR	Stone-free rate
TCTR	Thai Clinical Trials Registry
URS	Ureteroscopy
